# Less invasive surfactant administration combined with nasal high frequency oscillatory ventilation for an extremely low birth weight infant with severe hypercapnia: A case report

**DOI:** 10.1097/MD.0000000000022796

**Published:** 2020-10-16

**Authors:** Shanshan Pan, Zhiqun Zhang

**Affiliations:** a Department of Clinical Medicine, Hangzhou Medical College; b Department of Neonatology, Affiliated Hangzhou First People's Hospital, Zhejiang University School of Medicine, Hangzhou City, Zhejiang, China.

**Keywords:** hypercapnia, less invasive surfactant administration, nasal high frequency oscillatory ventilation, respiratory distress syndrome

## Abstract

**Background:**

Respiratory distress syndrome (RDS) is a common neonatal condition in premature infants. Its treatment often requires the use of surfactants. The administration of surfactants has evolved to less invasive surfactant administration (LISA) methods in recent years. Nasal high frequency oscillatory ventilation (nHFOV) is also a good new technology for respiratory support. The use of LISA combined with nHFOV for RDS has not been reported.

**Case summary:**

A 970 g male infant who was born at 29^+1^ weeks of gestational age suffered progressive dyspnea immediately after birth.

**Diagnosis:**

According to his clinical symptoms, X-ray, and blood gas analysis results, the extremely low birth weight infant was diagnosed with RDS and deep hypercapnic acidosis.

**Interventions:**

Less invasive surfactant administration combined with nasal high frequency oscillatory ventilation was utilized in the infant. The mean airway pressure (Paw) was set at 7 cm H2O, amplitude (ΔP) was set at grade 5.5 (level set according to the perception of vibration of the chest wall), frequency was set at 8 Hz, inspiratory time (Ti) was set at 33%, and FiO2 was set at 0.30.

**Outcomes:**

The patient's pCO2 dropped to 90.9 mm Hg in 2 hours and to 57.8 mm Hg in the following 4.5 hours; the patient was weaned from nHFOV after 12 hours. On day 61, the patient was discharged and free of respiratory symptoms.

**Conclusion:**

We speculate that less invasive surfactant administration combined with nasal high frequency oscillatory ventilation may be useful in the treatment of RDS with deep hypercapnia to avoid intubation.

## 1 Introduction

Respiratory distress syndrome (RDS) is a common neonatal condition in premature infants. Its treatment often requires the use of surfactants, which have been shown to reduce the risk of death and bronchopulmonary dysplasia (BPD).^[1]^ In recent years, the administration of surfactants has evolved from the INSURE method (intubation, surfactant administration and extubation) to less invasive surfactant administration (LISA) methods.^[2,3]^ The use of LISA combined with nasal high frequency oscillatory ventilation (nHFOV) for RDS has not been reported. We describe the use of LISA combined with nHFOV in an infant with severe RDS and deep hypercapnia.

## 2 Case summary

**Figure 1:**
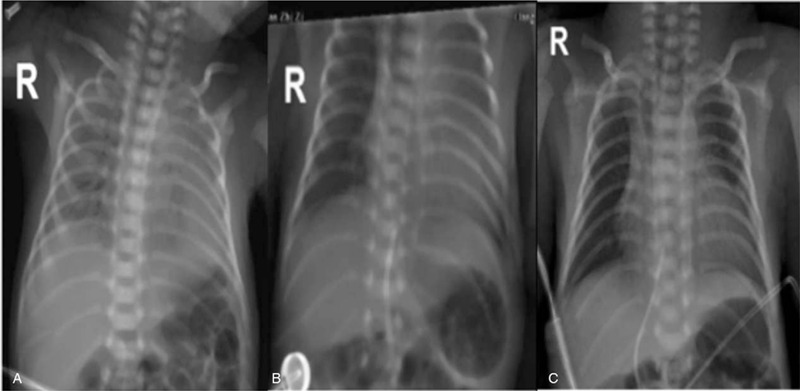
A: Initial chest radiograph showing white lung before surfactant. B: Chest radiograph two hours after surfactant showed a good improvement in lung translucency. C: Chest radiograph showed significant improvement four hours after nHFOV.

A 970 g male infant was born at 29^+1^ weeks of gestational age (GA) to a 34-year-old multipara mother by cesarean section owing to gestational diabetes mellitus. The mother had received a full course of antenatal steroids. The APGAR scores were 6 and 7 at the first and fifth minutes, respectively. The baby was given mask continuous positive airway pressure (CPAP) in the delivery room and was transferred to the NICU immediately. On admission, the blood gas of the newborn showed severe hypercapnia (pH 7.02, pCO_2_ 107.4 mm Hg). Approximately half an hour after birth, and chest X-ray (Fig. 1A) was compatible with RDS. Therefore, the patient was given surfactant by the less invasive surfactant administration (LISA) technique under nasal high frequency oscillatory ventilation (nHFOV) (CNO, Medin, Germany). A repeat chest X-ray showed an improvement in lung opacity (Fig. 1B/C) after surfactant administration; nHFOV was continued owing to CO2 retention. The ventilatory settings and blood gas parameters before and within 12 hours of nHFOV are shown in Table 1. The patient required nHFOV only for 12 hours, and then the CO_2_ retention completely vanished. Afterwards, the patient was ventilated with nCPAP for 7 days, and intubation was avoided. The transfontanelle ultrasound and MRI scan were normal. On day 61, the patient was discharged and free of respiratory symptoms.

**Table 1:**
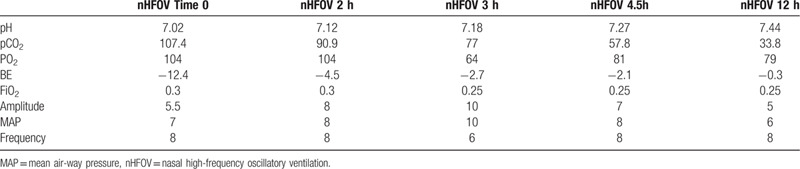
Data on respiratory support and arterial blood gas in the first 24 hours of nHFOV.

## 3 Discussion

Surfactant administration is a well-recognized management approach for RDS. The INSURE procedure (transient intubation for surfactant administration, followed by brief ventilation with final extubation to restore noninvasive respiratory support in spontaneously breathing preterm infants) is widely recommended.^[2]^ To reduce the potential risk of tracheal intubation and lung injury due to ventilation, even if for a short period during the INSURE procedure, LISA techniques have been described.^[4]^ Compared with traditional surfactant administration, spontaneous breathing is more conducive to the distribution of surfactant in the lungs.^[5]^

HFOV is a mode of mechanical ventilation that supports ventilation with small tidal volumes that are less than dead space at a supraphysiological respiratory frequency, providing constant lung expansion and effective removal of CO2.^[6]^ On the other hand, noninvasive ventilation has become increasingly and widely used in recent years because invasive mechanical ventilation is associated with BPD and impaired neurodevelopmental outcomes in preterm infants.^[7]^ Consequently, in recent years, several methods of noninvasive ventilation, including nCPAP, nasal intermittent positive-pressure ventilation, and high-flow nasal cannula, have been used with the hope of preventing endotracheal mechanical ventilation and BPD.^[8]^ Therefore, nHFOV could be a good combination of being noninvasive while allowing alveolar ventilation with constant lung expansion.

Furthermore, is it most beneficial to use the LISA technique on preterm neonates receiving nHFOV? There are few trials designed to test this hypothesis. However, some studies have provided evidence in support of the potential benefits.^[9,10]^ A recent study confirms that LISA strategies decrease the risks of the composite outcome of BPD or death and of early nCPAP failure when compared to “intubation-surfactant-extubation” approaches,^[9]^ and the latest European guideline recommends that LISA is the preferred mode of surfactant administration for spontaneously breathing babies on nCPAP for experienced clinicians.^[3]^ Our meta-analysis found that in preterm infants, the use of nHFOV rather than nCPAP was beneficial in terms of improved CO_2_ elimination and a reduced risk of intubation for mechanical ventilation.^[10]^ We speculate that surfactant administration with the LISA method in preterm neonates receiving nHFOV may have a theoretical rationale, and it may be feasible for the treatment of RDS in preterm infants.

In our case, the newborn's blood gas analysis after admission showed severe respiratory acidosis (PH: 7.02, pCO_2_: 107.4 mmHg), and he was definitively diagnosed with RDS. According to previous experience, newborns with blood gas showing severe hypercapnia (pH < 7.2, pCO_2_ > 65 mmHg) should be treated with conventional mechanical ventilation^[11]^ after surfactant administration. If treatment with conventional mechanical ventilation is not effective, HFOV should be used for remedial treatment. However, we did not use invasive mechanical ventilation and conventional surfactant administration techniques (INSURE) as reviews have recommended. Based on our experience and the above literature, we used nHFOV support combined with the LISA method instead of the conventional INSURE technique. What needs to be emphasized was that during the administration process, the respiratory support was nHFOV, not nCPAP, which was commonly used in the previous literature. We propose that using nHFOV support combined with surfactant administration with the LISA method is more beneficial than nCPAP combined with the LISA method. The mechanism may be as follows: the nHFOV mode can open the obliterated airways and alveoli; at the same time, the very fast ventilation frequency of nHFOV accelerates the distribution of exogenous surfactant in the small airways, and its unique gas exchange mode is also conducive to the uniform distribution of exogenous surfactant on the alveolar wall.

In our patient, after surfactant administration by the LISA method with nHFOV support, the partial pressure of carbon dioxide dropped progressively to 90.9 mmHg after 2 hours, to 77 mmHg after 3 hours, to 57.8 mmHg after 4.5 hours, and to 33.8 mmHg after 12 hours. nHFOV was stopped after 12 hours, nasal CPAP was supplied for a week, and a high flow cannula was supplied for an additional 4 weeks. We speculate that the previous criteria of intubation based on hypercapnia (pH < 7.2, pCO_2_ > 65 mmHg) were too loose. Furthermore, in our case, consequences such as intraventricular hemorrhage (grades III and IV), retinopathy of prematurity, and bronchopulmonary dysplasia at 36 weeks of postmenstrual age did not occur, which to some extent indicated that less invasive surfactant administration combined with nasal high frequency oscillatory ventilation might have fewer complications.

To the best of our knowledge, this is the first case report of surfactant administration with the LISA method in a preterm neonate who was receiving nHFOV support. Our case report suggests that nHFOV combined with LISA may significantly remove CO_2_ and avoid invasive mechanical ventilation. Therefore, we believe that a randomized controlled trial is urgently needed to confirm the effect and safety of LISA combined with nHFOV in extremely preterm infants.

## Author contributions

**Conceptualization:** Zhiqun Zhang.

**Funding acquisition:** Zhiqun Zhang.

**Investigation:** Shanshan Pan, Zhiqun Zhang.

**Writing – review & editing:** Shanshan Pan, Zhiqun Zhang.
